# Prognostic and immune correlation evaluation of a novel cuproptosis-related genes signature in hepatocellular carcinoma

**DOI:** 10.3389/fphar.2022.1074123

**Published:** 2022-12-14

**Authors:** Zheng Zhou, Yusong Zhou, Dongbo Liu, Qingping Yang, Mengjie Tang, Wei Liu

**Affiliations:** ^1^ Department of Otolaryngology Head and Neck, The Third Xiangya Hospital, Central South University, Changsha, China; ^2^ Department of Pharmacy, The Third Xiangya Hospital, Central South University, Changsha, China; ^3^ Department of Pharmacy, Zunyi Medical University, Zunyi, China; ^4^ Department of Pathology, Hunan Cancer Hospital, The Affiliated Cancer Hospital of Xiangya School of Medicine, Central South University, Changsha, China

**Keywords:** cuproptosis, HCC, prognostic, immune, mutation landscape

## Abstract

Hepatocellular carcinoma (HCC) is one of the world’s malignant tumors with high morbidity and mortality. Cuproptosis is a novel form of cell death. However, the prognostic evaluation and immune relevance of cuproptosis-related genes (CRGs) in HCC are largely unknown. In our study, we constructed a prognostic model of CRGs in HCC and performed immune infiltration, functional analysis, immune checkpoint and drug sensitivity analysis. Systematically elaborated the prognostic and immune correlation of CRGs in HCC. The results showed that 15 CRGs were up-regulated or down-regulated in HCC, and the mutation frequency of CRGs reached 10.33% in HCC, with CDKN2A having the highest mutation frequency. These 19 CRGs were mainly involved in the mitochondrion, immune response and metabolic pathways. Five selected genes (CDKN2A, DLAT, DLST, GLS, PDHA1) were involved in constructing a prognostic CRGs model that enables the overall survival in HCC patients to be predicted with moderate to high accuracy. Prognostic CRGs, especially CDKN2A, the independent factor of HCC prognosis, may be closely associated with immune-cell infiltration, tumor mutation burden (TMB), microsatellite instability(MSI), and immune checkpoints. CD274, CTLA4, LAG3, PDCD1, PDCD1LG2 and SIGLEC15 may be identified as potential therapeutic targets and CD274 correlated highly with prognostic genes. Quantitative Real-Time PCR (qRT-PCR) and immunohistochemical were performed to validate the mRNA and protein expression levels of CDKN2A in adjacent normal tissues and HCC tissues, and the results were consistent with gene difference analysis. In conclusion, CRGs, especially CDKN2A, may serve as potential prognostic predictors in HCC patients and provide novel insights into cancer therapy.

## 1 Introduction

Hepatocellular carcinoma (HCC), one of the most common malignant tumors of the digestive system, has the second highest mortality rate after colorectal cancer. Among new cases and deaths of HCC worldwide in 2020, 905,677 (4.7%) were newly diagnosed, and 83,0180 (8.3%) were newly diagnosed (Sung et al., 2021). According to the World Health Organization, more than 1 million patients will die of liver cancer in 2030, and the 5-year survival rate of liver cancer is less than 20%, second only to pancreatic cancer (Ren et al., 2021). Therefore, because of the high incidence rate and mortality of HCC, building a more effective prognostic model can provide useful evidence for the occurrence and development of HCC.

Copper is an indispensable trace element involved in various biological processes. Recent studies have shown that copper levels in serum and tumor tissue are significantly elevated in cancer patients compared to healthy patients (Ishida et al., 2013; Blockhuys et al., 2017; Ge et al., 2022). It has been found that dysregulation of copper homeostasis may cause cytotoxicity, and alterations in copper levels can cause the development and progression of cancer (Babak and Ahn, 2021). Many surveys have shown that the pathophysiological process of chronic liver disease is related to copper metabolism, and long-term bad habits of copper ion exposure may lead to chronic hepatotoxicity and ultimately cause liver cancer (Nakaichi et al., 2021; Liu et al., 2022). In addition, cuproptosis has recently attracted much attention in cancer research. Previous studies have concluded that cuproptosis can be a potential prognostic factor for patients with renal clear cell carcinoma and provide strategies for immunotherapy (Bian et al., 2022; Ji et al., 2022). Similarly, there have been some advances in the prognostic study of cuproptosis in tumors such as breast carcinoma and uterine corpus endometrial carcinoma (Chen, 2022; Sha et al., 2022). Although in previous studies, the prognostic characteristics and immune response of cuproptosis-related lncRNAs in HCC have also been investigated (Zhang et al., 2022a; Zhang et al., 2022b; Chen et al., 2022), and the prognostic value of CRGs in HCC has also been correspondingly investigated (Zhang et al., 2022c; Liu et al., 2022). However, the research on the relationship between CRGs and immunity needs further study.

Therefore, our study will investigate the relationship between CRGs and the prognosis and immunity of HCC, evaluate the constructed model, elucidate the importance of CRGs for HCC, and lay a certain clinical foundation for future treatment.

## 2 Methods and material

### 2.1 Datasets and preprocessing

On 15 July 2022, We downloaded the RNA sequencing (RNA-seq) data and the corresponding clinical information from 371 HCC patients using the Cancer Genome Atlas (TCGA) database, and the clinical information of HCC patients are shown in Supplementary Table S1. In addition, somatic cell datasets and Copy number variation (CNV) data for HCC have been downloaded from the TCGA database and the University of California Santa Cruz (UCSC) Xena websites. Data analysis was performed using R (version 4.0.3) and R Bioconductor packages. The expression data were normalized to transcripts per kilobase million (TPM) values before further analysis.

### 2.2 Identify differentially expressed CRGs

Based on previous studies (Polishchuk et al., 2019; Aubert et al., 2020; Dong et al., 2021; Ren et al., 2021; Bian et al., 2022; Chen, 2022; Kahlson and Dixon, 2022; Tsvetkov et al., 2022), a total of 19 CRGs (NFE2L2, NLRP3, ATP7B, ATP7A, SLC31A1, FDX1, LIAS, LIPT1, LIPT2, DLD, DLAT, PDHA1, PDHB, MTF1, GLS, CDKN2A, DBT, GCSH, DLST) were obtained. Differences in CRGs expression between HCC and adjacent normal tissues were identified using the “ggplot2” package. We then constructed a protein-protein interaction (PPI) network (the minimum required interaction score of 0.9) for 19 CRGs using the Search Tool for Retrieving Interacting Genes (STRING).

### 2.3 Mutation analysis of CRGs

Using the “maftools” package generated Mutation frequencies and oncoplot waterfall plots for 19 CRGs in HCC patients. Using the “RCircos” package in R, the locations of CNV alterations in 19 CRGs on 23 chromosomes were mapped.

### 2.4 Functional enrichment analysis

By http://vip.sangerbox.com/home.html, 19 CRGs were mapped in the Gene Ontology (GO) and the Kyoto Encyclopedia of Genes and Genomes (KEGG) analysis for enrichment analysis in HCC.

### 2.5 Assessment of prognostic characteristics associated with CRGs

A prognostic overall survival (OS) analysis was performed to identify potential prognostic characteristics. Log-rank tests were performed to calculate *p*-values, hazard ratios (HRs), and 95% confidence intervals. Based on these prognostic CRGs, a prognostic model was constructed using LASSO Cox regression analysis. The resulting model risk score was formulated as follows: risk score = exp-gene1 * coef-gene1 + exp-gene2 * coef-gene2+ …+ exp-genei * coef-genei. According to the median risk score, TCGA-HCC patients were divided into low-risk and high-risk subgroups. The Kaplan-Meier analysis compared the two subgroups’ overall survival (OS) time. Predictive accuracy and risk scores were assessed for each gene by performing a temporal receiver operating characteristic (ROC) analysis. Predictive nomograms were developed to predict overall survival at 1, 3, and 5 years.

### 2.6 Immune infiltration, TMB and MSI analysis

Then, we used the Tumor Immunity Estimation Resource (TIMER, https://cistrome.shinyapps.io/timer/), a web portal that comprehensively analyzes tumor-infiltrating immune cells to analyze the association between prognostic CRGs and immune infiltration. TIMER’s “Genes” module allows visualizing the correlation of gene expression with immune infiltrate levels in HCC. In the analysis of TMB and MSI, Spearman correlation analysis was used to calculate the correlation between gene expression and TMB and MSI scores, and *p* < 0.05 was considered statistically significant.

### 2.7 Risk model gene analysis

We analyzed the association of five prognostic genes with tumor grade and pTNM stage using the “ggplot2” package. HCC patients were divided into four phases for gene expression comparison and into early and late phases for comparison with adjacent normal tissues.

### 2.8 Immune-checkpoints analysis

We selected CD274, CTLA4, HAVCR2, LAG3, PDCD1, PDCD1LG2, TIGIT and SIGLEC15 as immune-checkpoint-relevant transcripts extracted the expression data of these eight genes to assess the expression of the immune checkpoints and co-expression-of-prognostic CRGs with these immune checkpoints.

### 2.9 Tissue samples

We collected 6 pairs of HCC tissues and adjacent normal tissues from the Third Xiangya Hospital of Central South University. These tissues were used to detect CDKN2A expression levels by qRT-PCR and immunohistochemistry. The Ethics Committee of the Third Xiangya Hospital of Central South University has approved the study. The approval number is I-22287.

### 2.10 Quantitative real-time PCR

To lyse cells, 1 ml Trizol reagent (TaKaRa, Japan) was added to the sample tissue according to the actual manufacturer’s instructions and incubated on a shaker for 15 min at room temperature. Total RNA was subsequently extracted from target samples. One microgram of RNA was reverse transcribed into cDNA using Revert Aid First Strand cDNA Synthesis Kit (Thermo, United States). Quantitative RT-PCR was then performed with Pro Taq HS Premix Probe qPCR Kit (Accurate, Hunan, China). The GAPDH gene was used as an endogenous control gene for normalizing the expression of target genes. Primers used in this study included CDKN2A(forward 5′-CCG​TGG​ACC​TGG​CTG​AGG​AG-3′, reverse 5′-CGG​GGA​TGT​CTG​AGG​GAC​CTT​C-3′), GAPDH (5′-CAG​GAG​GCA​TTG​CTG​AT-3′, 5′-GAA​GGC​TGG​GGC​TCA​TTT-3′).

### 2.11 Immunohistochemical staining

The tissues were sectioned and embedded in paraffin. Sections were incubated overnight at 4°C with anti-CDKN2A antibody (1:200 dilution; proteintech, China). Slides were washed with phosphate-buffered saline (PBS) and incubated with a goat anti-rabbit IgG secondary antibody conjugated with fluorescein isothiocyanate (ZSDB-BIO, China) for 30 min with washed slides. After washing with PBS, they were incubated with an antifade reagent (Invitrogen, United States). Staining was visualized to determine protein expression levels using an Olympus CX41 fluorescence microscope (Olympus, Japan). The analysis results were performed using ImageJ software.

## 3 Results

### 3.1 The different expression of CRGs of HCC and gene mutation

**FIGURE 1:**
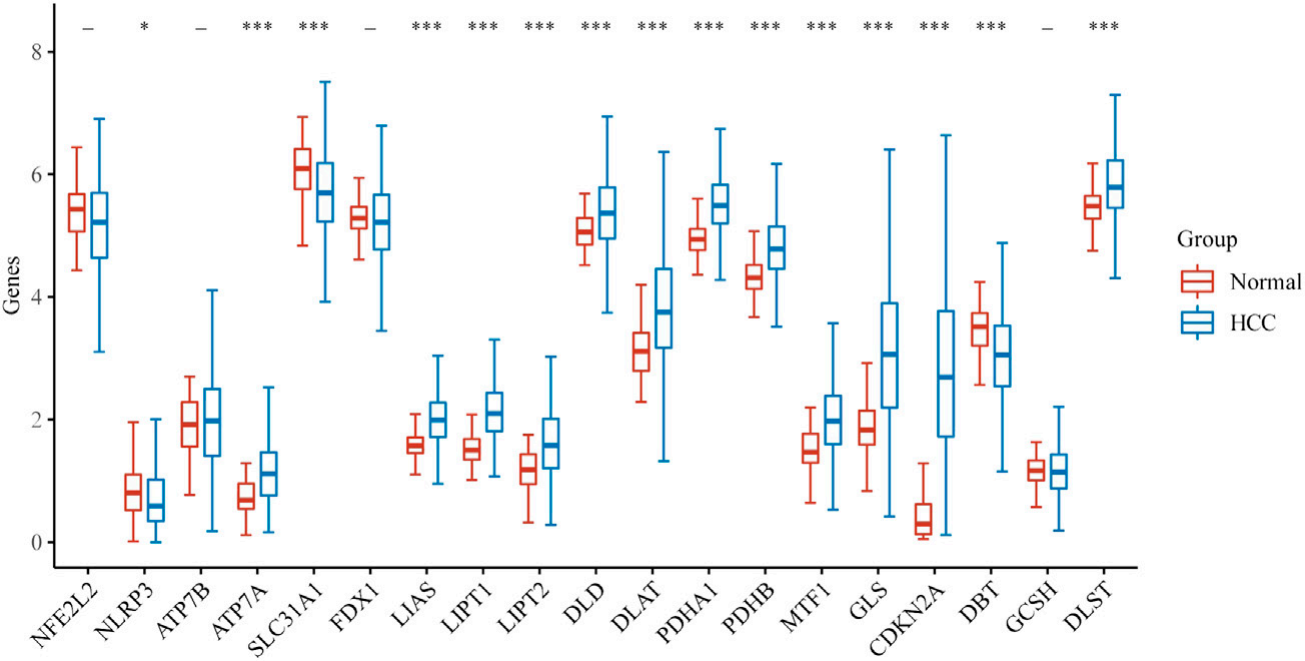
The expression of CRGs in HCC. (*p < 0.05, **p < 0.01, ***p < 0.001, asterisks (*) stand for significance levels).

We first explored the different expressions of 19 CRGs in HCC and the adjacent normal tissues by the UCSC Xena. In HCC, 15 CRGs were up-regulated or down-regulated (Figure 1). More specifically, compared to adjacent normal tissues, ATP7A, LIAS, LIPT1, LIPT2, DLD, DLAT, PDHA1, PDHB, MTF1, GLS, CDKN2A and DLST was increased, whereas the expression of NLRP3, SLC31A1, and DBT was decreased in HCC. A PPI analysis was constructed to detect the interaction of these CRGs, and the results showed that DLAT, DLST, PDHA1, DLD, and LIPT1 were central genes (Supplementary Figure S1A). Supplementary Figure S1B shows the correlation network containing all CRGs.

### 3.2 Genetic variation profile of CRGs in HCC

**FIGURE 2:**
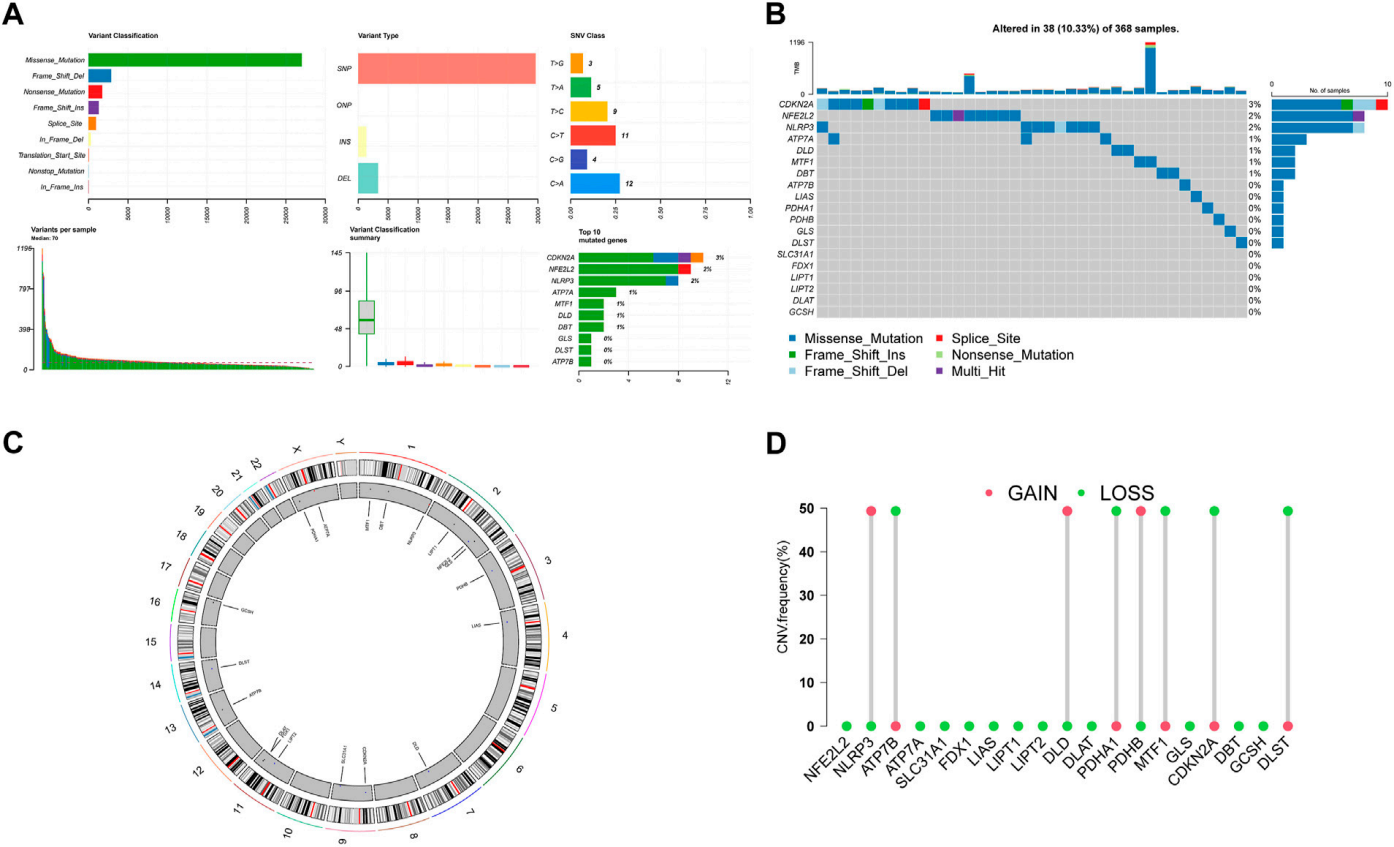
Landscape of genetic variation of CRGs in HCC **(A)**, **(B)** The mutation of frequency and classification of 19 CRGs in HCC. **(C)** The location of CNV alteration of 19 CRGs on 23 chromosomes in the HCC cohort. **(D)** The CNV variation frequency of 19 CRGs in the HCC cohort. The height of the column represented the alteration frequency.

Then we summarize the incidence of copy number variants and somatic mutations in 19 CRGs in HCC. Figures 2A, B showed 38 of 368 (10.33%) HCC samples showed gene mutations. Missense mutations were the most ordinary variant classification (Figure 2A). SNPs were the most ordinary variant type, and C > A was ranked as the top SNV category. Among 19 CRGs, CDKN2A had the highest mutation frequency, followed by NFE2L2 and NLRP3 (Figure 2B). Figure 2C demonstrates the location of CNV alterations on chromosomes for these 19 CRGs. We also investigated the frequency of CNV alterations and showed that these 19 CRGs showed prevalent CNV alterations. Among 19 CRGs, NLRP3, DLD, and PBHB had copy number amplification, while CNV deletion frequencies were prevalent for ATP7B, PDHA1, MTF1, CDKN2A, and DLST (Figure 2D).

### 3.3 Functional enrichment analysis of CRGs

**FIGURE 3:**
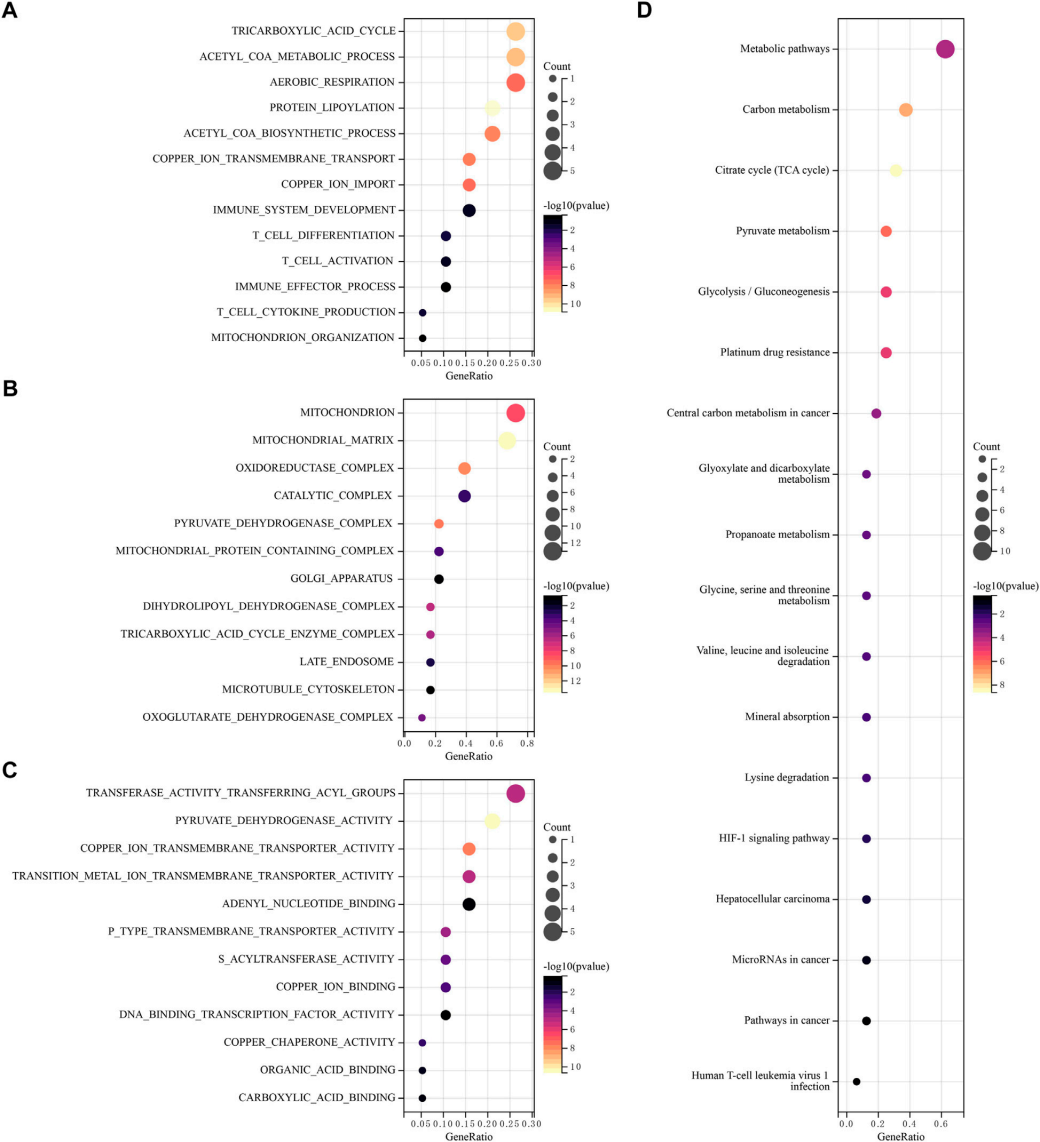
Functional enrichment analysis of CRGs. The enriched item in gene ontology analysis. **(A)** BP, **(B)** CC, **(C)** MF. **(D)** The enriched item in Kyoto Encyclopedia of Genes and Genomes analysis.

Using GO and KEGG databases, pathways were analyzed to reveal the function of CRGs. We found that GO was divided into three categories: biological pathway (BP), cytological component (CC), and molecular function (MF). In the BP, these 19 CRGs are mainly involved tricarboxylic acid cycle, acetyl COA metabolic process, aerobic respiration and immune response (Figure 3A). In the CC, these genes mainly participated in mitochondrion, oxidoreductase matrix, catalytic complex and pyruvate dehydrogenase complex (Figure 3B). In the MF, these genes mainly involved transferase activity transferring acyl groups, copper ion transmembrane transporter activity and DNA binding transcription factor activity (Figure 3C). In addition, KEGG pathway analysis showed that 19 CRGs have mainly participated in metabolic pathways, Glycolysis/Gluconeogenesis, Citrate cycle (TCA cycle), Platinum drug resistance and Human T-cell leukemia virus-1 infection (Figure 3D).

### 3.4 Construction of CRGs model

**FIGURE 4:**
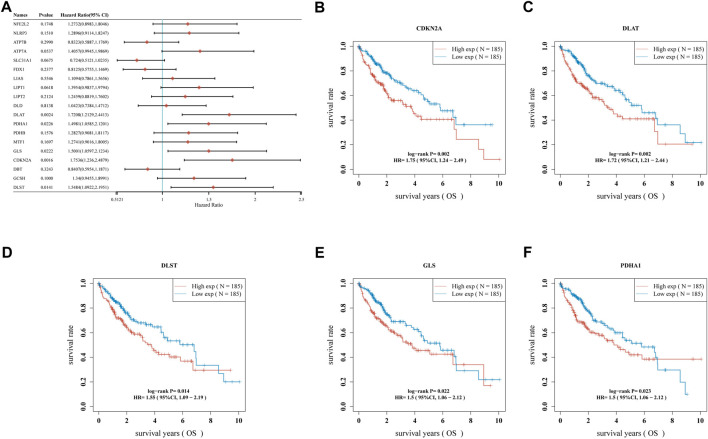
The prognostic value of CRGs in HCC. **(A)** Forest plot showing p-values for the 19 CRGs, **(B)** CDKN2A, **(C)** DLAT, **(D)** DLST, **(E)** GLS, **(F)** PDHA1 in HCC patients in the high-/low-expression group.

**FIGURE 5:**
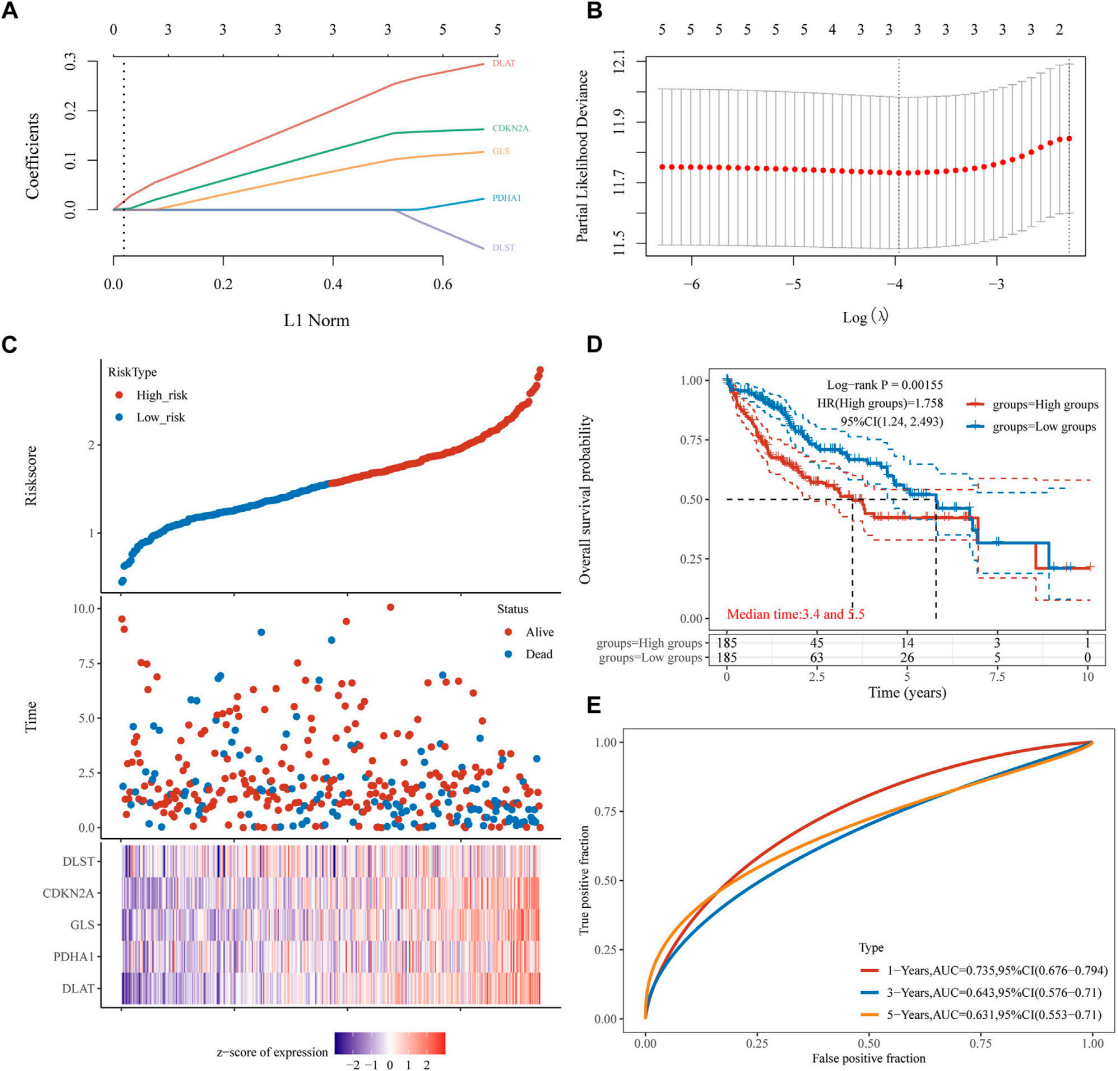
Construction of a prognostic CRGs model. **(A)** LASSO coefficient profiles of the five CRGs. **(B)** Plots of the ten-fold cross-validation error rates. **(C)** Distribution of risk score, survival status, and the expression of five prognostic CRGs in HCC. **(D)**, **(E)** Overall survival curves for HCC patients in the high-/low-risk group and the ROC curve for measuring the predictive value.

A univariate Cox regression analysis was performed to find CRGs with a prognostic value, and five CRGs were screened. As a result, the forest plot showed *p*-values for the 19 CRGs (Figure 4A). The results showed that the low expression group of HCC patients with CDKN2A (Figure 4B, *p* = 0.002), DLAT (Figure 4C, *p* = 0.002), DLST (Figure 4D, *p* = 0.014) and GLS (Figure 4E, *p* = 0.022) and PDHA1 (Figure 4F, *p* = 0.023) had better survival than the high expression group. A five-gene model was constructed according to the optimum λ value obtained by LASSO Cox regression analysis (Figures 5A, B). Risk score was calculated as Risk score = (0.1645) * CDKN2A + (0.3033) * DLAT + (-0.096) * DLST + (0.1196) * GLS + (0.0289)* PDHA1. Based on the median score, the distribution of risk scores and survival status, patients in the training set were randomly divided into high and low-risk groups, and the distribution of risk scores, survival status, and expression of these five genes are shown in Figure 5C, with patients having an increased risk of death and a decreased survival time as the risk score increased. Kaplan-Meier curves showed that high-risk HCC patients had a lower overall survival probability than low-risk patients (Figure 5D, *p* = 0.00155), and 1, 3, and 5 years ROC curves were showed AUCs of 0.735, 0.643, and 0.631, respectively (Figure 5E).

### 3.5 Constructing the prediction nomogram

**FIGURE 6:**
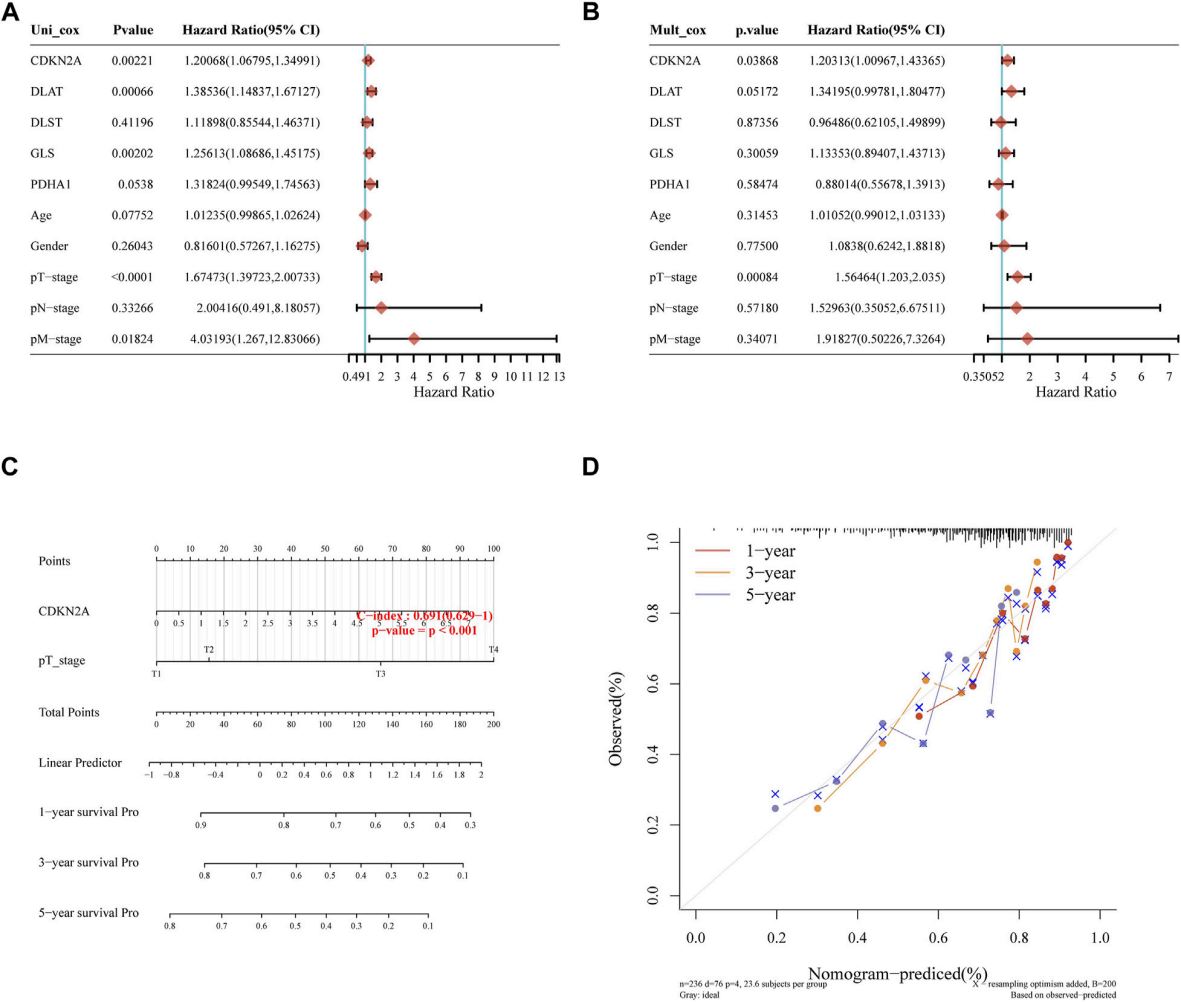
Constructing the predictive nomogram. **(A)**, **(B)** Hazard ratio and *p*-value of the constituents involved in univariate and multivariate Cox regression considering clinical parameters and five prognostic CRGs in HCC. **(C)** The nomogram predicted the 1-year, 3-year, and 5-year overall survival rates of HCC patients. **(D)** Calibration curve for the overall survival nomogram model in the discovery group.

Later, we constructed a predictive nomogram to predict the survival probability. The five prognostic CRGs and the clinicopathological features were considered in the nomogram. CDKN2A expression and pT stage were found they be independent factors that could affect the prognosis of HCC patients in univariate and multivariate analysis (Figures 6A, B). Predicted nomograms indicated that 1-year, 3-year and 5-year OS curves could be predicted relatively well compared to the ideal model in the entire cohort (Figures 6C, D).

### 3.6 The correlation between tumor immune infiltration and CRGs in HCC

**FIGURE 7:**
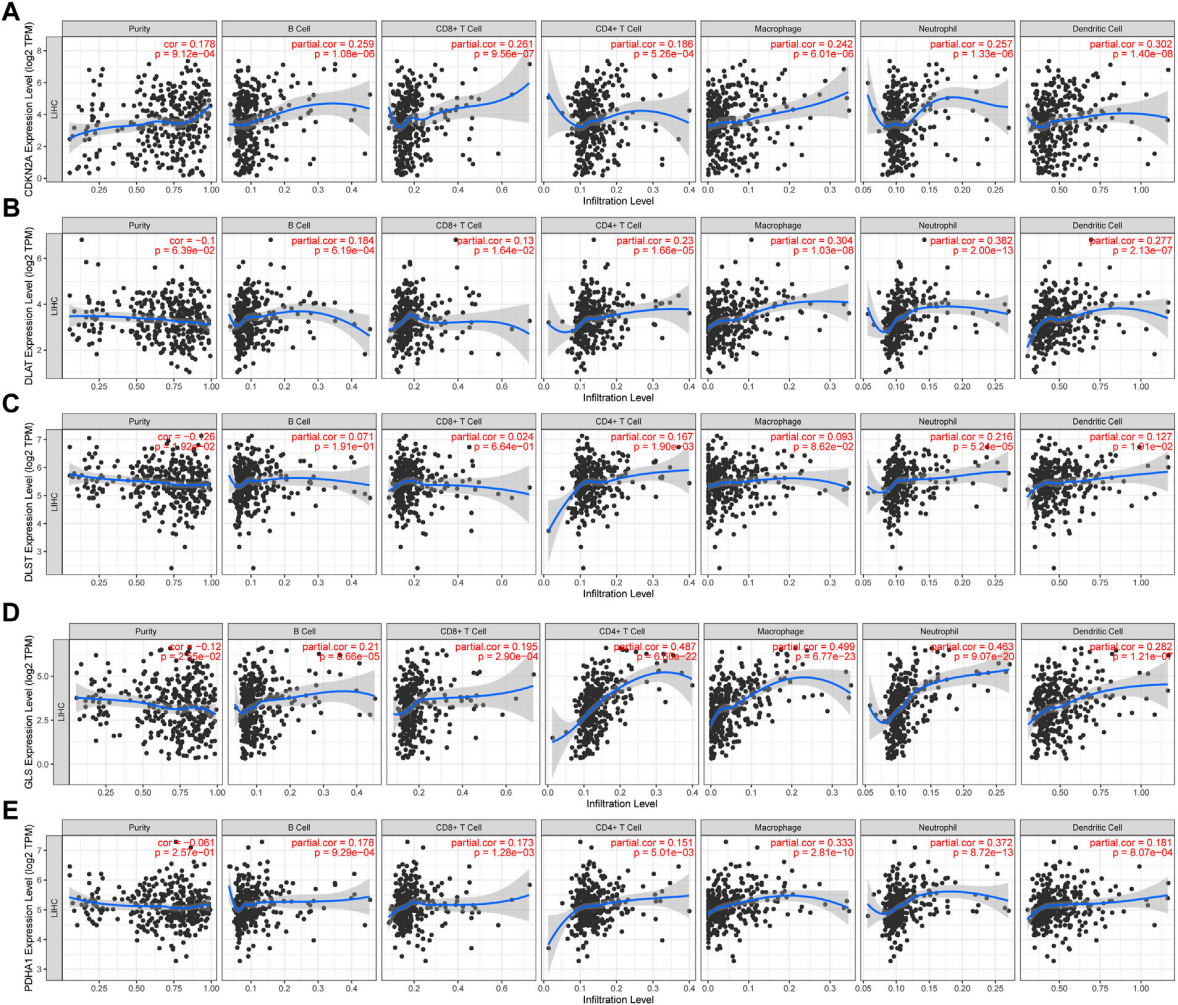
The correlation between five prognostic CRGs and immune infiltration. The association between the abundance of immune cells and the expression of CDKN2A **(A)**, DLAT **(B)**, DLST **(C)**, GLS **(D)**, and PDHA1 **(E)** in HCC.

In developing the tumor’s immune microenvironment, cuproptosis plays a crucial role. Our study also elucidated the association between the expression of prognostic CRGs (DLAT, PDHA1, GLS, CDKN2A, DLST) and HCC immune infiltration using the TIMER database. The results showed that CDKN2A expression was positively associated with the expression of B cells (*p* = 1.08e-06), CD8^+^ T cells (*p* = 9.56e-07), CD4^+^ T cells (*p* = 5.26e-04), neutrophils (*p* = 1.33e-06), macrophages (*p* = 6.01e-06), and medullary dendritic cells (*p* = 1.40e-08) (Figure 7A). DLAT expression was positively associated with the expression of B cells (*p* = 6.19–04), CD8^+^ T cells (*p* = 1.64e-02), CD4^+^ T cells (*p* = 1.66e—05), neutrophils (*p* = 2.00e—13), macrophages (*p* = 1.03e-08), and medullary dendritic cells (*p* = 2.13e-07) (Figure 7B). DLST expression was positively associated with the expression of B cells (*p* = 1.91e-01), CD8^+^ T cells (*p* = 6.64e-01), CD4^+^ T cells (*p* = 1.90e-03), neutrophils (*p* = 5.24e-05), macrophages (*p* = 8.62e-02), and medullary dendritic cells (*p* = 1.91e-02) (Figure 7C). Moreover, GLS expression was also positively associated with the expression of B cells (*p* = 8.66e-05), CD8^+^ T cells (*p* = 2.90e-04), CD4^+^ T cells (*p* = 6.60e-22), neutrophils (*p* = 9.07e-20), macrophages (*p* = 6.77e-23), and medullary dendritic cells (*p* = 1.21e-07) (Figure 7D). PDHA1 expression was positively associated with the expression of B cells (*p* = 9.29e-04), CD8^+^ T cells (*p* = 1.28e-03), CD4^+^ T cells (*p* = 5.01e-03), neutrophils (*p* = 8.72e-13), macrophages (*p* = 2.81e-10), and medullary dendritic cells (*p* = 8.07e-04) (Figure 7E). This study suggests a significant association between CRGs and tumor-immune infiltration.

### 3.7 Risk model gene analysis

**FIGURE 8:**
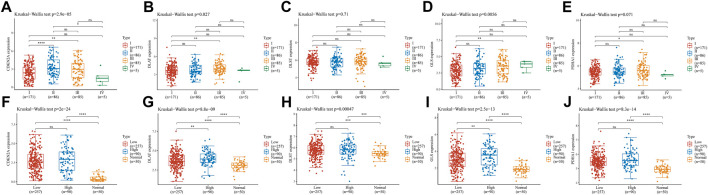
Risk model gene analysis **(A–E)** The expression of different pTNM between five CRGs in HCC. CDKN2A **(A)**, DLAT **(B)**, DLST **(C)**, GLS **(D)**, and PDHA1 **(E) (F–J)** The expression of low and high tumor-grades and adjacent normal tissues between five CRGs in HCC. CDKN2A **(F)**, DLAT **(G)**, DLST **(H)**, GLS **(I)**, and PDHA1 **(J)**.

We analyzed the association of prognostic genes with tumor grade and pTNM stage. The expression of CDKN2A (Figures 8A, *p* = 2.9e-05), DLTA (Figure 8B, *p* = 0.027), GLS (Figure 8D, *p* = 0.0056) was significantly different at the pTNM stage in HCC. In addition, CDKN2A (Figure 8F, *p* = 2e-24), DLAT (Figure 8G, *p* = 8.8e-09), DLST(Figure 8H, *p* = 0.00047), GLS(Figure 8I, *p* = 2.5e-13) and PDHA1 (Figure 8J, *p* = 8.3e-14) were significantly different at the in HCC patients with high tumor grades, low tumor grades and adjacent normal tissues.

### 3.8 TMB, MSI analysis of CRGs

**FIGURE 9:**
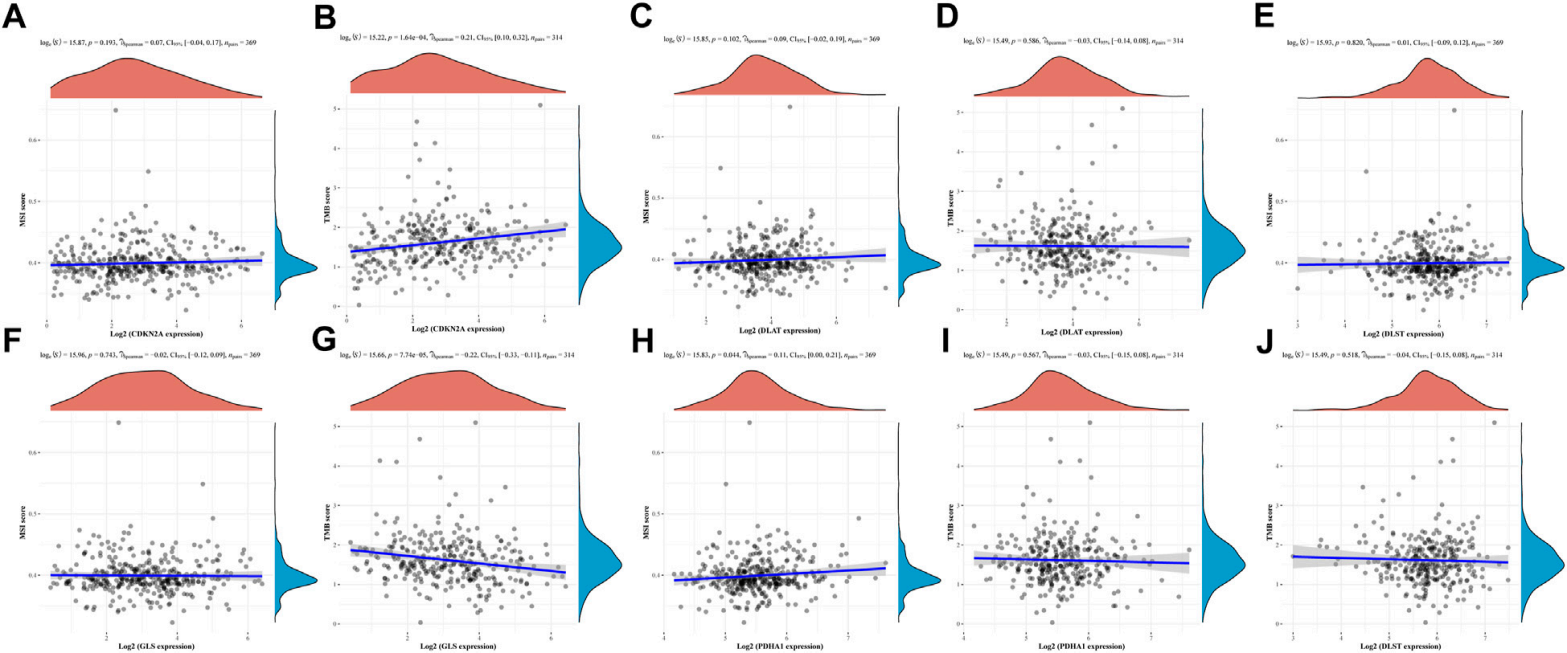
TMB, MSI analysis of CRGs in HCC **(A,C,E,F,H)** Correlation between five prognostic CRGs and TMB. **(B,D,G,I,J)** Correlation between five prognostic CRGs and MSI.

The above results showed that CRGs were significantly associated with tumor immune infiltration. Subsequently, we analyzed the relationship between CRGs and TMB and MSI, which could further elucidate whether CRGs could serve as a biomarker for drug screening and lay the foundation for subsequent studies. The results showed a positive correlation between MSI and PDHA1 (Figure 9H, *p* = 0.044) but no significant correlation between MSI and CDKN2A, DLAT, DLST and GLS (Figures 9A, C, E, F). Meanwhile, TMB was positively correlated with CDKN2A (Figure 9B, *p* = 1.64e-4) and negatively correlated with GLS (Figure 9G, *p* = 7.74e-05) but not significantly associated with DLAT, DLST, PDHA1 (Figures 9D, J, I).

### 3.8 Drug sensitivity and immune checkpoint analysis in HCC

**FIGURE 10:**
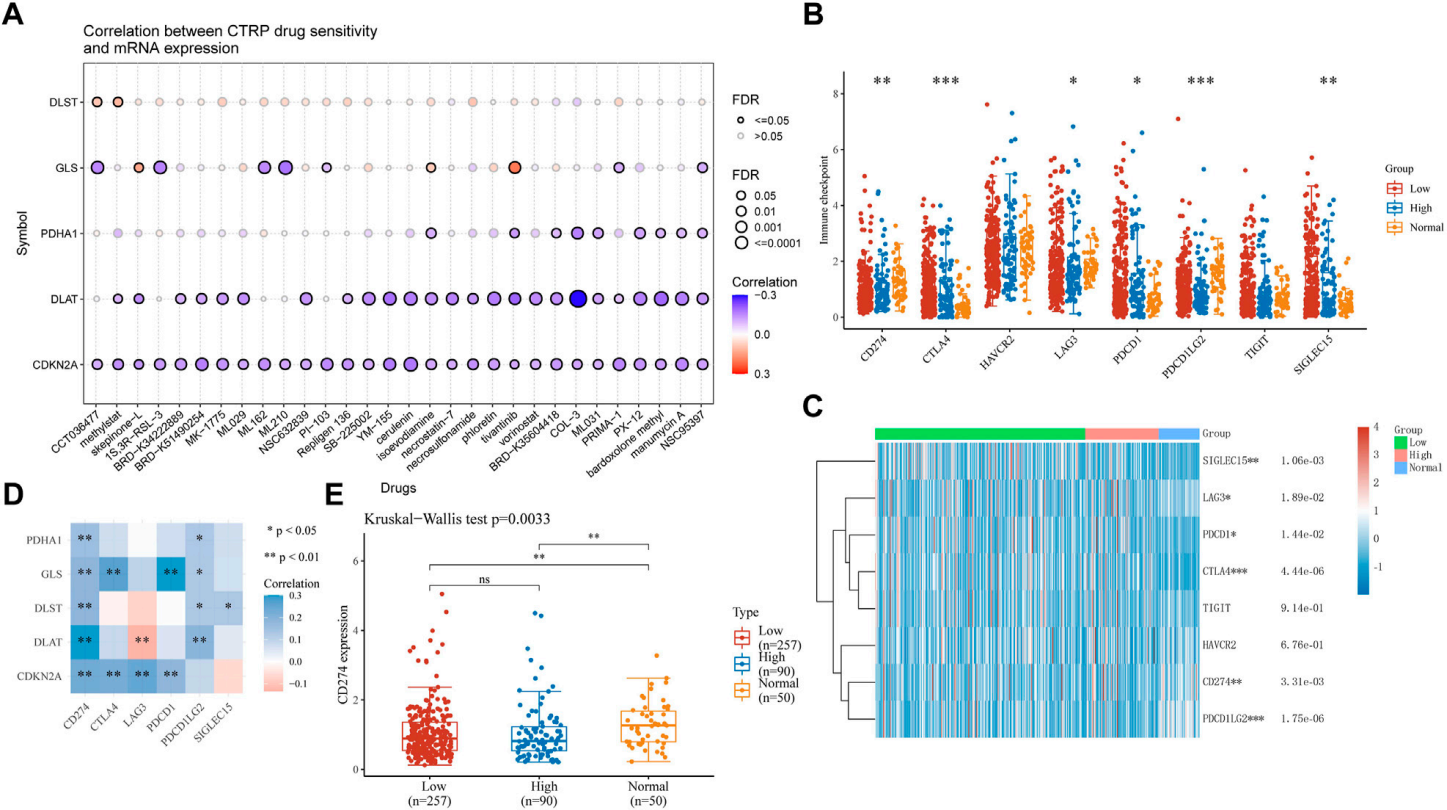
Drug sensitivity and immune checkpoints analysis **(A)** Drug-sensitivity analysis of five prognostic CRGs in HCC. **(B)**, **(C)** Different expressions of immune-checkpoints in low-, high-grade and adjacent normal tissue in HCC. (*p < 0.05, **p < 0.01, ***p < 0.001, asterisks (*) stand for significance levels.) **(D)** The correlation analysis between five prognostic CRGs and immune checkpoints. **(E)** The expression of CD274 in low-, high-grade and adjacent normal tissue in HCC. (*p < 0.05, **p < 0.01, ***p < 0.001, asterisks (*) stand for significance levels.).

Drug therapy is an important means of cancer treatment. In this study, drug sensitivity analysis and immune checkpoint analysis provide a certain basis for the mechanism of drug therapy. In drug sensitivity analysis, the expression of CDKN2A, DLAT, PDHA1, and GLS was negatively associated with some or most drugs in the Cancer Therapy Response Portal database, and the expression of DLST positively correlated (Figure 10A). HCC samples were divided into high-risk and low-risk groups and compared with adjacent normal tissues for immune checkpoint analysis. The expression of CD274, CTLA4, LAG3, PDCD1, PDCD1LG2 and SIGLEC15 was significantly different between adjacent normal tissues and tumor tissues (Figures 10B, C). Then, we performed a correlation analysis between five CRGs with prognostic value and immune checkpoint genes. We found that PDHA1 and CD274, PDCD1LG2, GLS were significantly correlated with CD274, CTLA4, PDCD1, PDCD1LG2, DLST and CD274, PDCD1LG2, SIGLEC15, DLAT and CD274, PDCD1LG2, CDKN2A was significantly correlated with CD274, CTLA4, LAG3 and PDCD1, while DLTA was negatively correlated with LAG3 (Figure 10D). Based on the positive correlation between CD274 and all five prognosis-related genes, we performed another analysis of CD274 in HCC and showed a significant difference between high-risk and low-risk HCC and adjacent normal tissues (Figure 10E, *p* = 0.0033).

### 3.10 Validation of CDKN2A expression in HCC

**FIGURE 11:**
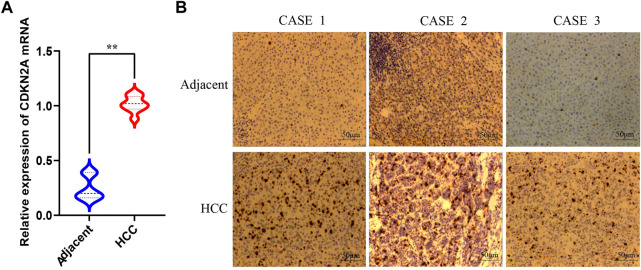
qRT-PCR and immunohistochemical of CDKN2A **(A)** Relative expression of CDKN2A mRNA in HCC and adjacent normal tissues. **(B)** Immunohistochemical of CDKN2A protein in HCC and adjacent normal tissues.

qRT-PCR was performed to validate CDKN2A expression levels in HCC and adjacent normal tissues. The results showed the expression level of CDKN2A in HCC was significantly higher than that in adjacent normal tissues (Figure 11A), which was consistent with the results of gene difference analysis. The protein expression level of CDKN2A was subsequently determined by immunohistochemical staining, and the results were consistent with the expression level of mRNA (Figure 11B).

## 4 Discussion

HCC accounts for the majority of primary liver cancers. Globally, liver cancer is the fourth most common cause of cancer-related death and ranks sixth among incident cases (Villanueva, 2019). There has been no increase in mortality and incidence of HCC incidence-based (IB) in the US population in the past 15 years, the first in 40 years. The insignificant increase in morbidity and mortality from IB suggests that HCC incidence and mortality tend to stabilize and approach a peak (Njei et al., 2015). Therefore, effective preventive measures are the key to effectively controlling the incidence of HCC. Recently, it has been found that cuproptosis is a novel programmed cell death that plays an important role in the occurrence and development of malignant tumors, so it is crucial to study the significance of CRGs in the development and prognosis of HCC.

By gene expression profiling, the results showed that ATP7A, LIAS, LIPT1, LIPT2, DLD, DLAT, PDHA1, PDHB, MTF1, GLS, CDKN2A, and DLST were up-regulated, while NLRP3, SLC31A1, and DBT were down-regulated in HCC compared to adjacent normal tissues, which is consistent with previous studies. In previous studies, CDKN2A was highly mutated and expressed in advanced HCC, which may be related to the pathogenesis of HCC (Khemlina et al., 2017; Luo et al., 2021), but no other gene is expressed and functional in HCC. Correlation analysis showed that most CRGs were positively associated with each other. In previous studies, GLS and PDHA1 were found to play a synergistic role in promoting glutamine dependence in prostate cancer patients (Li et al., 2016). In addition, PDHA1, PDHB, DLAT, and DLD also play a synergistic role in pyruvate dehydrogenase complex deficiency (Inui et al., 2022).

In GO enrichment, acetyl coenzyme A (acetyl COA) is the main raw material of the tricarboxylic acid (TCA) cycle, and CRGs are involved in the acetyl COA metabolic process, which has been demonstrated that copper directly binds to the fatty acylation component of the TCA cycle, resulting in toxic protein stress and ultimately cell death (Tsvetkov et al., 2022). TCA flux is regulated reciprocally by TCA intermediates’ replenishment and removal processes. Although present as a minor activity in other tissues, replenishment and replenishment of hepatocytes are highly active in hepatocytes, and their balance is critical for the function of the TCA cycle (Linehan et al., 2019). In MF, these genes are also involved in the function of DNA binding transcription factor activity. The green regulation of transcription factors (TFs) can regulate protein expression levels, and the basic biochemical response in TF regulation is the binding of TFs to regulatory DNA (Gao et al., 2021). A study has developed a database named hTFtarget by creating a comprehensive TF-target relationship library for humans, which can serve as a useful resource for researchers in TF regulation and gene expression communities (Zhang et al., 2020), and cancer cell lines can be identified using gene enrichment analysis and gene expression data (Zhang et al., 2021). T cells can mediate the killing of HCC and cause T cell failure in cancer (Hung et al., 2021; Hong et al., 2022). Thus, the expression of CRGs can impact the immune process.

Further prognostic studies showed that DLAT, PDHA1, GLS, CDKN2A, and DLST were potential prognostic biomarkers in HCC. Another study also showed that CDKN2A promoter methylation is associated with enhanced HCC risk and plays a key role in the progression of HCC (Zhou et al., 2018); GLS can promote cancer cell metabolism and biogenesis (Huang et al., 2019), and its differential expression can regulate the prognosis of human cancer (Saha et al., 2019); pyruvate dehydrogenase E1α subunit (PDHA1) is one of the main functional enzymes in energy metabolism, and has been considered to be closely related to tumor development and progression (Sun et al., 2019). In addition, we performed and verified prognostic features associated with cuproptosis by LASSO Cox regression analysis, including these five prognostic biomarkers (DLAT, PDHA1, GLS, CDKN2A, and DLST), which performed well in predicting the prognosis of HCC patients. To our knowledge, the TNM system is the most dependable method for predicting the prognosis of HCC patients. Although the TNM system has already been elaborated to have some prognostic value in the survival analysis of HCC (Chen et al., 2021), there is still a great lack of these prognostic characteristics for assessing clinical HCC patients. The current study identifies the first cuproptosis-related prognostic signature in HCC patients that can be used clinically to predict the prognosis of HCC patients.

In immune infiltration analysis, the results showed that the expression of CDKN2A, DLAT, DLST, GLS and PDHA1 was positively associated with the abundance of certain immune cells, including CD8^+^ T cells and CD4^+^ T cells. Recently, similar results have emerged in immune infiltrates concerning CRGs in other types of cancer. Previous studies have demonstrated that CDKN2A expression is positively associated with the level of infiltration of immune cells in HCC (Luo et al., 2021). The expression of CDKN2A showed a significant association with the level of immune infiltration in clear cell renal cell carcinoma (Bian et al., 2022). The expression of CDKN2A and GLS is also positively associated with the abundance of certain immune cells in endometrial cancer (Chen, 2022).

In the analysis of TMB and MSI, we found a significant association between PDHA1 and MSI, CDKN2A and TMB. Similar studies have previously seen a significant association between CDKN2A and TMB (Chen, 2022; Jiang et al., 2022). However, the association between PDHA1 and MSI is the first to appear in our study, which may become an important research direction to control the development of HCC.

In drug sensitivity analysis and immune checkpoint analysis, the results showed that CDKN2A, DLAT, PDHA1 and GLS were negatively associated with some or most drugs in the cancer therapeutic response portal database, and the expression of DLST was positively correlated. CDKN2A has also demonstrated drug relevance in cancer treatment response portals in previous studies (He et al., 2020). Still, the relevance of the expression of several other genes in some or most drugs in cancer treatment response portals is unclear. This study further illustrates the significance of the expression of DLAT, DLST, GLS, and PDHA1 for cancer treatment. Expressions of CD274, CTLA4, LAG3, PDCD1, PDCD1LG2, and SIGLEC15 were shown to be significantly different between normal and tumor samples in immune checkpoint analysis. Increased expression of programmed cell death 1 (PD-L1, also known as CD274) has previously been reported in HCC treated with sorafenib (El Dika et al., 2019), indicating that CD274 expression can effectively assess the therapeutic effect of HCC during the treatment of HCC.

qRT-PCR and immunohistochemical were performed to validate the mRNA and protein expression levels of CDKN2A in adjacent normal tissues and HCC tissues. The results were consistent with gene difference analysis. CDKN2A is a prognostic biomarker that is associated with immune infiltration in HCC, and its expression can contribute to the regulation of tumor-associated macrophages (Luo et al., 2021). CDKN2A promoter methylation is associated with an increased risk of HCC (Zhou et al., 2018), so CDKN2A has some value as a prognostic gene.

However, our study has many shortcomings. First, the risk profile of CRGs was performed based on data obtained in public databases, which is inevitably limited by inherent case selection bias, and larger prospective and multicenter clinical studies are needed to confirm our findings. Secondly, although prognostic scores focusing on the expression signature of CRGs showed good performance in predicting HCC prognosis, some other important genes with predictive value were not considered in this study. Finally, some mechanistic experimental studies *in vivo* and *in vitro* are needed to validate our results further.

## 5 Conclusion

To summarize, we accomplished a comprehensive and systematic bioinformatics analysis of HCC patients by constructing prognostic models for CRGs and immune assessment. Five selected genes (CDKN2A, DLAT, DLST, GLS, PDHA1) were involved in constructing a prognostic CRGs model that enables the overall survival in HCC patients to be predicted with moderate to high accuracy. Prognostic CRGs, especially CDKN2A, the independent factor of HCC prognosis, may be closely correlated with immune-cell infiltration, TMB, MSI, and immune checkpoints. The qRT-PCR and immunohistochemical staining results verified the mRNA and protein expression levels of CDKN2A in adjacent normal tissues and HCC tissues. CD274, CTLA4, LAG3, PDCD1, PDCD1LG2 and SIGLEC15 may be identified as potential therapeutic targets and CD274 correlated highly with prognostic genes. However, due to the limitations of this study, further studies should be performed to validate this finding.

## Data Availability

The original contributions presented in the study are included in the article/Supplementary Material, further inquiries can be directed to the corresponding author.
