# Supplementary material for: Prognostic and immune correlation evaluation of a novel cuproptosis-related genes signature in hepatocellular carcinoma
Source: Front Pharmacol. 2022 Dec 14;13:1074123. doi: 10.3389/fphar.2022.1074123 (PMC9795230; doi:10.3389/fphar.2022.1074123)
Supplement: Supplementary file 1 [file Table1.DOCX]

Supplementary Material

**Supplementary Figure S1.** (A) protein-protein interaction (PPI) of CRGs. (B) the correlation network contains all CRGs.

**Supplementary Table S1.** The clinical characteristics of LIHC patients in the TCGA cohort.

| Clinical characters | Number |
| --- | --- |
| Gender  Male  Female | 241  130 |
| Age |  |
| Mean (SD) | 59.4 (13.5) |
| Median [MIN, MAX] | 61 [16,90] |
| TNM stage  I  II  III  IV | 171  86  85  5 |
| pT_stage  T1  T2  T3  T4  TX | 181  94  80  13  1 |
| pN_stage  N0  N1  NX | 252  4  114 |
| pM_stage  M0  M1  MX | 266  4  101 |
