# Supplementary figures and images for: Prognostic and immune correlation evaluation of a novel cuproptosis-related genes signature in hepatocellular carcinoma
Source: Front Pharmacol. 2022 Dec 14;13:1074123. doi: 10.3389/fphar.2022.1074123 (PMC9795230; doi:10.3389/fphar.2022.1074123)

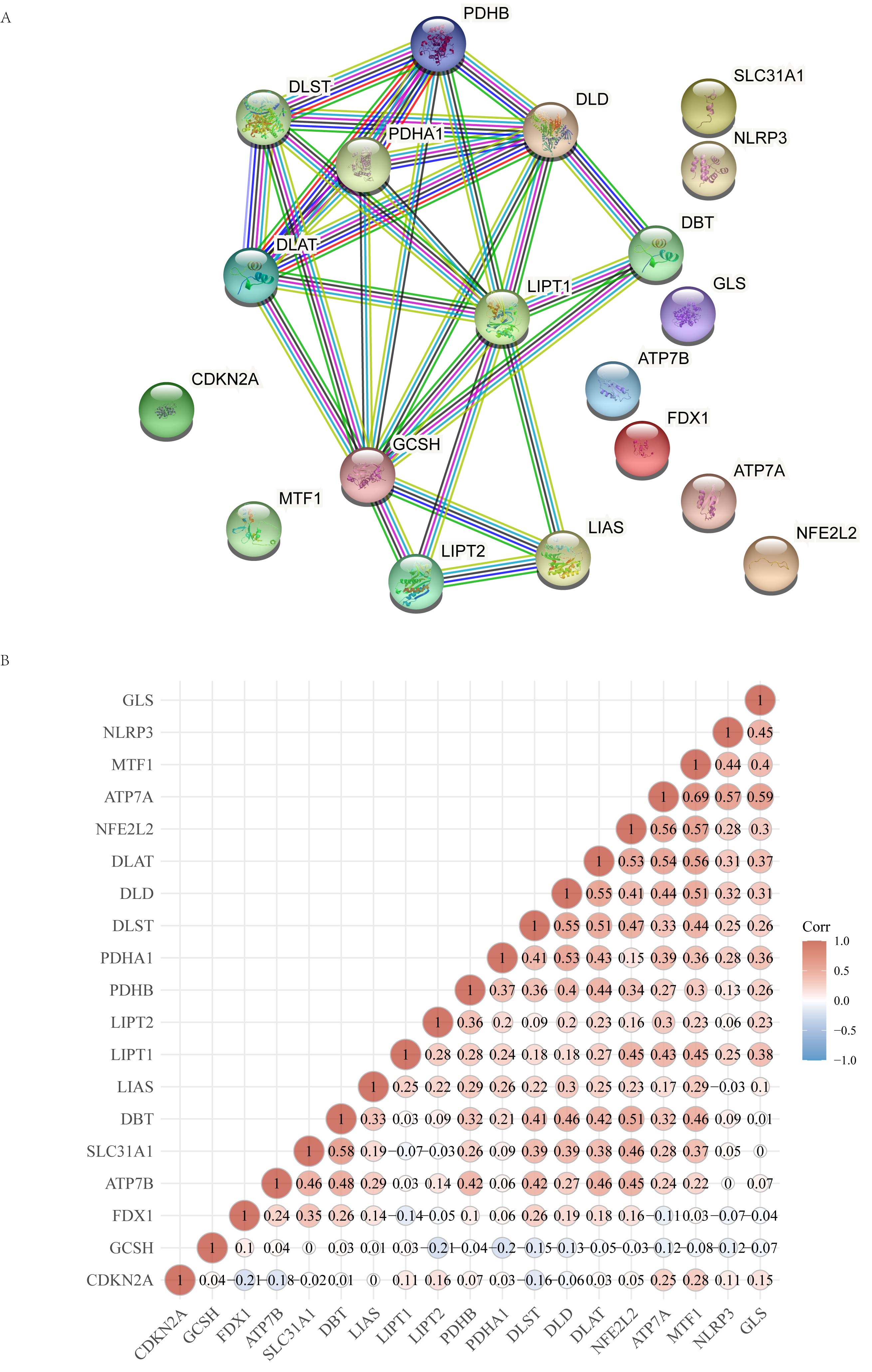

Supplement: Supplementary file 2 [file Image1.JPEG]
